# Outcomes and complications of sponges versus tires for scleral buckling in primary rhegmatogenous retinal detachment: The Manchester Buckle Study

**DOI:** 10.1111/aos.70082

**Published:** 2026-01-27

**Authors:** Peter Kiraly, Myrta Lippera, Naseer Ally, Ritu Agarwal, George Moussa, Tsveta Ivanova, Felipe Dhawahir‐Scala, Niall Patton, George Turner, Stephen Charles, Assad Jalil, Kirti M. Jasani

**Affiliations:** ^1^ Manchester Royal Eye Hospital Manchester University Hospitals NHS Foundation Trust Manchester UK; ^2^ Oxford Eye Hospital Oxford University Hospitals NHS Foundation Trust Oxford UK; ^3^ Nuffield Laboratory of Ophthalmology University of Oxford Oxford UK; ^4^ Department of Ophthalmology Humanitas Mater Domini Castellanza Varese Italy; ^5^ Division of Ophthalmology, Department of Neurosciences University of the Witwatersrand Johannesburg South Africa; ^6^ Barnet Hospital, Royal Free London NHS Foundation Trust Barnet UK

**Keywords:** best‐corrected visual acuity, explant, explant extrusion, rhegmatogenous retinal detachment, scleral buckling, silicone sponge, silicone tire, single‐surgery anatomical success

## Abstract

**Purpose:**

To compare preoperative characteristics and postoperative anatomical and functional outcomes of scleral buckle surgery using sponges versus tires, as well as explant‐related complications and the frequency of explant removal.

**Methods:**

This retrospective study included patients with primary rhegmatogenous retinal detachment (RRD) who underwent primary scleral buckling at the Manchester Royal Eye Hospital between 2008 and 2023. Preoperative data included age, macula status, type of RRD, ocular comorbidities and best‐corrected visual acuity (BCVA). Postoperative outcomes included single‐surgery anatomical success (SSAS), BCVA and explant‐related complications (extrusion, infection, high intraocular pressure and persistent diplopia).

**Results:**

Among 562 patients (mean age 36 ± 12 years), 183 received sponges and 379 received tires. Sponges were more commonly used in dialysis‐related RRD (54% vs. 10%; *p* < 0.01) and macula‐off eyes (52% vs. 36%; *p* < 0.01), and associated with worse preoperative BCVA (logMAR 0.84 ± 0.81 vs. 0.57 ± 0.72; *p* < 0.01). SSAS rates were similar between groups (86.0% vs. 83.4%; *p* = 0.44) and mean BCVA improvement (ΔBCVA) did not differ significantly (0.39 ± 0.57 vs. 0.29 ± 0.58 logMAR; *p* = 0.29). In multivariable analysis adjusting for relevant clinical covariates, explant type was not independently associated with postoperative BCVA (*B* = –0.10; *p* = 0.28) or SSAS (aOR = 0.79; *p* = 0.71). Buckle extrusion occurred more frequently in the sponge group (12.6% vs. 3.4%; *p* < 0.01), leading to higher explant removal rates (14.8% vs. 6.1%; *p* < 0.01).

**Conclusions:**

Sponges were preferentially used in dialysis‐related RRD cases and were associated with worse preoperative BCVA and a higher proportion of macula‐off RRDs. SSAS rates and ΔBCVA improvement were similar between groups. Sponges had significantly higher extrusion rates, resulting in more frequent explant removal.

## 1 INTRODUCTION

Scleral buckling remains an important surgical option for patients with rhegmatogenous retinal detachment (RRD), despite a growing trend towards pars plana vitrectomy (PPV) over the past decades (Ramulu et al., 2010). It is now commonly used in younger, phakic, myopic patients and in RRD without posterior vitreous detachment (PVD) as well as for atrophic breaks, inferior breaks and retinal dialysis (Fallico et al., 2022). In phakic patients with simple or moderately complex RRD, scleral buckling is associated with comparable anatomical outcomes and better functional outcomes compared to vitrectomy (Heimann et al., 2007; Safadi et al., 2021; Starr et al., 2021), with superior visual outcomes in macula‐off eyes undergoing prompt surgery (Baudin et al., 2021).

Although scleral buckle surgery is generally safe, it still carries a risk of ocular morbidity and can lead to significant postoperative complications (Papakostas & Vavvas, 2018). The most common refractive change is a myopic shift due to increased axial length, and its degree depends on the buckle indentation and the presence of the crystalline lens (Sato et al., 2003; Smiddy et al., 1989). Scleral buckle surgery can lead to extraocular infection in about 0.5%–5.6% of cases (Hahn et al., 1979; Lincoff et al., 1970; Russo & Ruiz, 1971). Early infections are typically caused by intraoperative contamination, whereas late infections are more often associated with secondary infection of an extruded implant (Russo & Ruiz, 1971). Historically, scleral abscesses were reported in about 4% of cases (Lincoff et al., 1965); however, their incidence has decreased significantly and is now very rare (Folk et al., 1987). Buckle extrusion through the conjunctiva can cause redness and pain and is one of the most common reasons for buckle removal (Moisseiev et al., 2017). Scleral buckle surgery can cause non‐perfusion of the anterior or posterior ciliary arteries, which can very rarely lead to anterior or posterior segment ischaemia (Papakostas & Vavvas, 2018). Diplopia following scleral buckle surgery has been described in approximately 3.8% of cases, with the most common cause being mechanical muscle restriction (Goezinne et al., 2012). Intraocular pressure (IOP) elevation was reported in 15 out of 52 eyes (28.9%) within 1 month of scleral buckle surgery (Mansoori et al., 2021). One study also reported the development of postoperative cystoid macular oedema in 20.7% of cases after scleral buckling for primary RRD (Gebler et al., 2022).

To date, a wide range of synthetic and natural materials have been tested for scleral buckling, with permanent silicone explants considered the gold standard. Silicone explants are well tolerated by ocular tissues due to their chemical inertness, long‐term in vivo stability and excellent biocompatibility (Baino, 2010; Brown et al., 2006; Schepens & Acosta, 1991). Currently, solid and sponge silicone implants are mostly used in clinical practice. Among solid silicone implants, silicone tires are the most commonly used and are easy to place; although they remain soft and pliable, their moulded geometry can create scleral indentation of the desired shape (Baino, 2010). Sponge silicone implants are also widely used for scleral buckling. Modern sponge designs come in a variety of shapes and thicknesses, allowing targeted indentation while minimizing the risks of extrusion, infection and ocular motility disturbances (Baino, 2010).

In this retrospective study, we compared preoperative characteristics, single‐surgery anatomical success (SSAS) rates and best‐corrected visual acuity (BCVA) following scleral buckle surgery for RRD using tire versus sponge silicone explants. We also assessed postoperative complication rates in each group and examined which explant type was more frequently removed.

## 2 METHODS

This retrospective study included patients from the Manchester Royal Eye Hospital who underwent scleral buckling between 2008 and 2023. This study adhered to the tenets of the Declaration of Helsinki and was registered as a clinical audit and service evaluation, with institutional approval obtained; therefore, formal Research Ethics Committee approval was not required in accordance with UK Health Research Authority guidance, as only anonymized routinely collected data were used. An electronic database search at the Manchester Royal Eye Hospital was conducted to identify RRD patients who underwent scleral buckle surgery as the primary procedure. In line with local clinical practice at Manchester Royal Eye Hospital, primary scleral buckling was generally reserved for non‐PVD RRD, whereas PPV was the preferred approach for PVD‐associated RRDs.

Surgery involved cryopexy of the retinal breaks or dialysis, followed by placement of a silicone tire or sponge explant sutured to the sclera with 5–0 non‐absorbable nylon sutures. Under indirect ophthalmoscopic visualization, the posterior edge of the explant was positioned at least 1 mm posterior to the most posterior retinal break. The choice between a sponge and a tire explant was made at the surgeon's discretion based on intraoperative assessment of retinal detachment morphology. In general, sponges were preferentially selected for dialysis‐related RRDs to provide a higher and more anterior indentation, whereas tires were more commonly used for retinal hole–related RRDs where a broader, more posterior support was preferred. The decision to perform external drainage was at the surgeon's discretion and, when undertaken, was performed in the area of maximal subretinal fluid using either a prang drain or a scleral cut‐down technique. Anterior chamber paracentesis was performed in the majority of cases at the surgeon's discretion to reduce intraocular pressure.

In our analysis, we collected demographic characteristics, preoperative RRD and functional characteristics, ocular comorbidities, intraoperative management, postoperative anatomical success and BCVA outcomes, postoperative complications related to the explant and management of explant‐related postoperative complications. Collected demographic data included gender and age at the time of surgery. RRD characteristics included type of RRD (dialysis‐related or retinal break–related). We recorded the presence of high myopia—defined as a spherical‐equivalent refractive error ≤−6.00 D or an axial length >26.0 mm—and any history of significant recent trauma to the operated eye. Preoperative BCVA was collected and converted to logMAR values for statistical analysis. Surgical details included the surgeon's grade (consultant, fellow or registrar) and the type of explant used. Postoperative SSAS, final anatomical success and BCVA were determined for all eyes as well as for the macula‐on and macula‐off subgroups. SSAS was defined as complete retinal reattachment following the primary scleral buckle, without the need for further reattachment surgery. Final anatomical success was defined as complete reattachment after the final vitreoretinal procedure, achieved without silicone oil tamponade. Collected postoperative explant‐related complications included extrusion, infection, high IOP and persistent diplopia (>6 months). Management of high IOP and persistent diplopia was also noted. The number of patients requiring explant removal was recorded, along with the reason for removal (e.g. extrusion, infection, slipped buckle, distortion, pain or conjunctival breakdown).

Statistical analyses were conducted using SPSS v20 (IBM Corp., Armonk, NY, USA). Normality for continuous data was assessed with the Shapiro–Wilk test. Continuous variables with a normal distribution were compared using Student's *t*‐test (two groups) or one‐way ANOVA (three or more groups). BCVA was non‐normally distributed and was therefore compared using the Mann–Whitney *U* test. Categorical variables were analysed with chi‐squared tests or Fisher's exact test when any expected cell count <5. Multivariable analyses were performed to adjust for potential confounders: a binary logistic regression model for SSAS and a multiple linear regression model for postoperative BCVA. Model fit was assessed using the Hosmer–Lemeshow test and Nagelkerke *R* ^2^ for logistic regression and the *F*‐test, adjusted *R* ^2^ and variance inflation factor (VIF) for linear regression. All tests were two‐tailed, and *p* < 0.05 was considered statistically significant.

## 3 RESULTS

### 3.1 Preoperative characteristics

We included 562 eyes: 183 received a sponge (32.6%) and 379 a tire (67.4%). Age and surgeon grade were similar between groups (*p* = 0.37 and *p* = 0.49). Tires were used more often in macula‐on RRD (64% vs. 48%; *p* < 0.01). Regarding RRD type, dialysis was present in 99/183 eyes (54%) in the sponge group versus 37/379 (10%) in the tire group (*p* < 0.01). Intravitreal air or gas was used in 14 eyes, including air in nine cases, expansile SF_6_ in four cases and expansile C_3_F_8_ in one case. A 360° encircling band was used in 10 eyes, all within the tire explant subgroup; 7 procedures were performed by the same surgeon, 8 eyes were macula‐on at presentation, 9 had retinal hole–related RRD (and 1 dialysis), 5 were highly myopic, SSAS was achieved in 9 eyes, and no explants required removal. Sponges were used exclusively as segmental buckles and were not employed as 360° encircling elements. Preoperative BCVA (logMAR) was significantly better in the tire group (0.57 ± 0.72) than in the sponge group (0.84 ± 0.81; *p* < 0.01). This difference was also seen in both the macula‐on subgroup (0.11 ± 0.22 in the tire group and 0.20 ± 0.25 in the sponge group; *p* < 0.01) and the macula‐off subgroup (1.05 ± 0.75 in the tire group and 1.29 ± 0.76 in the sponge group; *p* = 0.01). Full preoperative characteristics are shown in Table 1.

**TABLE 1:** Preoperative characteristics of a cohort of patients treated with sponge or tire explant.

Characteristic	Sponge (*n* = 183)	Tire (*n* = 379)	*p*‐value
Age (years, mean ± SD)	37 ± 13	36 ± 11	0.37
Surgeon grade			
Fellow (%)	137 (75%)	271 (71.5%)	0.49
Consultant (%)	44 (24%)	106 (28%)	
Registrar (%)	2 (1%)	2 (0.5%)	
Macula status			
Macula‐on (%)	88 (48%)	242 (64%)	**<0.01**
Macula‐off (%)	95 (52%)	137 (36%)	
Type of retinal detachment			
Dialysis (%)	99 (54%)	37 (10%)	**<0.01**
Hole (%)	84 (46%)	342 (90%)	
Ocular comorbidities			
High myopia (%)	14 (8%)	70 (18.0%)	**<0.01**
Trauma (%)	18 (10%)	13 (3.4%)	**<0.01**
Preoperative BCVA (all eyes)	0.84 ± 0.81	0.57 ± 0.72	**<0.01**
Preoperative BCVA (macula‐on)	0.20 ± 0.25	0.11 ± 0.22	**<0.01**
Preoperative BCVA (macula‐off)	1.29 ± 0.76	1.05 ± 0.75	**0.01**

*Note*: A *p*‐value of <0.05 was considered statistically significant (in bold).

Abbreviations: BCVA, best‐corrected visual acuity; SD, standard deviation.

### 3.2 Morphological and functional outcomes

SSAS was achieved in 157/183 eyes (86.0%) in the sponge group and 316/379 (83.4%) in the tire group (*p* = 0.44). Across all eyes, postoperative BCVA (logMAR) was better with tires (0.23 ± 0.43) than sponges (0.39 ± 0.45; p < 0.01). Among macula‐on eyes, SSAS was 80/88 (91%) with sponges and 203/242 (84%) with tires (*p* = 0.11); postoperative BCVA was similar (0.18 ± 0.35 vs. 0.12 ± 0.27; *p* = 0.15). For macula‐off eyes, SSAS was 77/95 (81%) with sponges and 114/137 (83.2%) with tires (*p* = 0.67); postoperative BCVA was better with tires (0.44 ± 0.56 vs. 0.59 ± 0.45; *p* = 0.03). In all eyes, the mean BCVA improvement (ΔBCVA) was 0.39 ± 0.57 in the sponge group and 0.29 ± 0.58 logMAR in the tire group (*p* = 0.29). In the sponge group, 26 of 183 eyes (14.2%) required a secondary surgical procedure. Of these, 9 eyes (35%) underwent a secondary scleral buckle and 17 eyes (65%) underwent secondary PPV. The mean interval between the primary and secondary procedures was 6.2 months. Overall, 25 patients underwent two surgeries, and 1 patient underwent three surgeries. Final anatomical success was achieved in all eyes in the sponge group. In the tire group, 63 of 379 eyes (16.6%) required re‐intervention. Among these, 20 eyes (32%) underwent a secondary scleral buckle and 43 eyes (68%) underwent secondary PPV. The mean interval between the primary and secondary procedures was 13.3 months. Overall, 60 patients underwent two surgeries, 2 patients underwent three surgeries, and 1 patient underwent four surgeries. Final anatomical success was achieved in 375 of 379 eyes (99%). Table 2 presents the anatomical and functional postoperative outcomes in both the sponge and tire groups.

**TABLE 2:** Postoperative outcomes of a cohort of patients treated with sponge and tire.

Outcome	Sponge (*n* = 183)	Tire (*n* = 379)	*p*‐value
SSAS (all eyes)	157 (86.0%)	316 (83.4%)	0.44
Postoperative BCVA (all eyes), mean ± SD	0.39 ± 0.45	0.23 ± 0.43	**<0.01**
BCVA improvement (all eyes), mean ± SD	0.39 ± 0.57	0.29 ± 0.58	0.29
SSAS (macula‐on)	80 (91%)	203 (84%)	0.11
Postoperative BCVA (macula‐on), mean ± SD	0.18 ± 0.35	0.12 ± 0.27	0.15
SSAS (macula‐off)	77 (81%)	114 (83.2%)	0.67
Postoperative BCVA (macula‐off), mean ± SD	0.59 ± 0.45	0.44 ± 0.56	**0.03**

*Note*: A *p*‐value of <0.05 was considered statistically significant (in bold).

Abbreviations: BCVA, best‐corrected visual acuity; SD, standard deviation; SSAS, single surgery anatomical success.

In multivariable logistic regression adjusting for age, macula status, type of RRD, preoperative BCVA, myopia, trauma and surgeon grade, explant type was not an independent predictor of SSAS (aOR = 0.79, 95% CI 0.22–2.80; *p* = 0.71). The model showed excellent calibration (Hosmer–Lemeshow *χ* ^2^ = 1.56, df = 8, *p* = 0.99) and modest explanatory power (Nagelkerke *R* ^2^ = 0.09). In multivariable linear regression adjusting for preoperative BCVA, macula status, RRD type, age, SSAS, trauma and myopia, explant type was not independently associated with postoperative BCVA (*B* = −0.10, SE = 0.09; *p* = 0.28). The multivariable model explained approximately 46% of the variance in postoperative BCVA (adjusted *R* ^2^ ≈ 0.46), with preoperative BCVA being the strongest predictor.

In the sponge group, 25 eyes (14%) received a 3 mm sponge, 86 (47%) a 4 mm and 72 (39%) a 5 mm. SSAS was 76%, 91% and 83%, respectively (*p* = 0.13). Postoperative logMAR BCVA was 0.26 ± 0.36, 0.39 ± 0.41 and 0.42 ± 0.50, with no significant difference (*p* = 0.34). Tire type was documented in 359 of 379 eyes in the tire group, with the tire type not specified in 20 eyes. Among the 359 tire eyes, 174 (48.5%) received a 277 tire, 105 (29.2%) a 287 tire and 80 (22.3%) a 276 tire. SSAS was 83.3%, 86.7% and 82.5%, respectively, with no significant difference (*p* = 0.69). Postoperative logMAR BCVA did not differ significantly among tire types (*p* = 0.12).

### 3.3 Postoperative complications

Mean follow‐up was 3.27 ± 2.15 years in the tire group and 4.29 ± 3.23 years in the sponge group (*p* < 0.01). In the tire group, 13/379 eyes (3.4%) developed buckle extrusion and 1 (0.3%) developed infection. High IOP occurred in 30 (7.9%); 3 (0.8%) underwent glaucoma surgery. Persistent diplopia occurred in 16 (4.2%), with 2 (0.5%) requiring strabismus surgery. Buckle removal was performed in 23 eyes (6.1%): 1 (0.3%) for infection, 7 (1.8%) for extrusion, 8 (2.1%) for diplopia, 5 (1.3%) for undocumented reasons and 2 (0.5%) for distortion. In the sponge group, extrusion occurred in 23/183 eyes (12.6%), and no infections were recorded. Elevated IOP occurred in 15 (8.2%) and was managed conservatively in all cases. Persistent diplopia occurred in 8 (4.4%), and 2 (1.1%) underwent strabismus surgery. Buckle removal was required in 27 eyes (14.8%): 13 (7.1%) for extrusion, 4 (2.2%) for diplopia, 2 (1.1%) for a slipped buckle, 2 (1.1%) for distortion, 1 (0.5%) for pain, 1 (0.5%) for conjunctival breakdown and 4 (2.2%) for undocumented reasons. Explant‐associated complication rates are shown in Table 3, and extrusion and buckle removal were significantly more frequent with sponges (both *p* < 0.01).

**TABLE 3:** Explant‐associated complications in the sponge and tire cohorts.

Variable	Sponge (*n* = 183)	Tire (*n* = 379)	*p*‐value
Follow‐up (years), mean ± SD	4.29 ± 3.23	3.27 ± 2.15	**<0.01**
Buckle extrusion, *n* (%)	23 (12.6)	13 (3.4)	**<0.01**
High IOP, *n* (%)	15 (8.2)	30 (7.9)	0.91
Persistent diplopia, *n* (%)	8 (4.4)	16 (4.2)	0.93
Buckle removal, *n* (%)	27 (14.8)	23 (6.1)	**<0.01**

*Note*: A *p*‐value of <0.05 was considered statistically significant (in bold).

Abbreviations: IOP, intraocular pressure; SD, standard deviation.

## 4 DISCUSSION

Silicone sponges and tires are both widely used in everyday clinical practice, but each has advantages and disadvantages and is often chosen according to the surgeon's preference. Sponges are soft and compressible, allowing easier intraoperative manipulation and potentially less postoperative discomfort. Also, because of their compressibility, sponges generally create a higher indent than tires. However, sponges are porous and can absorb fluid over time, which diminishes the indent in the long run. Sponges can also harbour bacteria, leading to a higher risk of infection and extrusion compared with solid implants. Tires, on the other hand, have a more stable indent over time and are less prone to infection and extrusion, yet they provide a shallower indent and might cause more postoperative discomfort and more ocular motility issues (Baino, 2010).

Non‐PVD RRDs are associated with dialysis and/or retinal holes and account for about 10% of primary RRDs. These RRDs are usually shallow (93%) and progress slowly (Rohowetz et al., 2023; Ung et al., 2005). In our study, age at presentation and surgeon grade distribution were similar in the sponge and tire groups. In the sponge group, about half of the eyes had retinal dialysis RRD and half had retinal hole RRD. By contrast, in the tire group, 90% of patients had retinal hole, and only 10% had dialysis. Eyes in the sponge group were more frequently associated with trauma than those in the tire group, reflecting the higher incidence of dialysis RRD, which is known to be associated with trauma (Rohowetz et al., 2023). In the sponge group, which included about half of the dialysis RRD cases, the reported trauma rate was 10%, which is lower compared with the 61.4% trauma rate reported in a retinal dialysis RRD cohort (Rohowetz et al., 2023). On the other hand, high myopia was more common in the tire group, which is a known risk factor in retinal hole RRD (Ung et al., 2005). Therefore, our findings suggest that sponges were used more frequently in patients with dialysis RRD, whereas tires were more commonly used in RRD cases with retinal holes with or without myopia. Sponges are more commonly used in dialysis RRD as they provide a higher, more anterior indent, closer to the dialysis. The sponge group had more macula‐off RRD cases than the tire group, resulting in poorer preoperative BCVA in the sponge group. Moreover, the worse preoperative BCVA observed in macula‐on eyes within the sponge group may be related to the higher incidence of trauma in these patients.

SSAS in all eyes was similar in both groups (86% in the sponge group vs. 83% in the tire group), which also closely aligns with the 86% rate reported by Salabati et al. (2024) following scleral buckling. Previous series of scleral buckling for primary RRD have largely reported overall anatomical and functional outcomes without stratifying results by explant type. Therefore, the higher indentation achieved by sponges (Baino, 2010) did not translate into better SSAS. However, it should be noted that there were more macula‐off RRD cases in the sponge group. Postoperative BCVA in all eyes was worse in the sponge group—likely because that group had worse preoperative BCVA—yet both groups showed similar postoperative BCVA improvements. For macula‐on eyes, SSAS did not differ significantly (91.0% in the sponge group vs. 84% in the tire group; *p* = 0.11). The numerical advantage for sponge may relate to the higher prevalence of dialysis RRD, which has been associated with favourable SSAS rates (Rohowetz et al., 2023), but this was not statistically confirmed in our study. Expectedly, postoperative BCVA among macula‐on eyes did not change substantially in either group. Meanwhile, among macula‐off eyes, SSAS was similar in both groups; however, postoperative BCVA was worse in the sponge group, again most likely due to poorer preoperative BCVA. There is a wide variety of silicone sponges and tires used for scleral buckle surgeries (Baino, 2010). In our study, both SSAS and postoperative BCVA were similar among the different silicone sponges, and the same applied for the various tires. Deep learning algorithms have shown promise in predicting late recurrence after scleral buckling (Catania et al., 2024) for RRD and may, in the future, help determine the optimal explant to achieve the best anatomical and functional outcomes.

In our study, only one patient in the tire group and none in the sponge group developed an infection, which is significantly lower than the previously reported range of 0.5–5.6% (Hahn et al., 1979; Lincoff et al., 1970; Russo & Ruiz, 1971). Moreover, our findings do not support earlier suggestions that sponges pose a higher infection risk due to their porosity, which could potentially facilitate bacterial growth (Lorenzano et al., 2007). Elevated IOP was observed at a similar rate in both groups and was consistent with the reported incidence of 5.4% in the literature (Lv et al., 2015). The majority of cases were managed conservatively, with three patients in the tire group requiring glaucoma surgery. Persistent diplopia was observed in 4.4% of cases in the sponge group and 4.2% in the tire group, which is slightly higher than the 2.7% previously reported by Lv et al. (2015). Our findings do not align with those of Patel et al. (2024), who reported more diplopia in solid silicone explants compared to sponges. In our study, the incidence of extrusion was higher in the sponge group than in the tire group. This increased rate of extrusion was the primary reason more patients in the sponge group required buckle removal. Specifically, 14.8% of sponge explants had to be removed compared to 6.1% in the tire group. A significant difference in follow‐up duration was observed between the groups, with longer follow‐up in the sponge group compared with the tire group. This discrepancy may have contributed to the higher observed rates of extrusion and explant removal in the sponge cohort, as longer follow‐up provides greater opportunity for late complications to manifest. Therefore, the comparison of long‐term complication rates between groups should be interpreted with some caution. Overall, the follow‐up time in this study was relatively short—4.29 years in the sponge group and 3.27 years in the tire group—compared to the average of 12.3 years to buckle removal reported by Patel et al. (2024). Consequently, it is possible that additional explants in our study will require removal as follow‐up continues.

To the best of our knowledge, this is the largest real‐world cohort comparing indications for various scleral explants, anatomical and functional outcomes with sponge versus tire buckles and explant‐related complications, including removal rates. Key limitations include the retrospective, single‐centre design, incomplete/missing data and unequal follow‐up durations between groups. The choice between sponge and tire was not randomized and was influenced by surgeon preference, with a tendency in our unit to use sponges in cases of dialysis‐related RRD, introducing a potential selection bias.

In conclusion, in our centre, sponges were more frequently selected for retinal detachments associated with dialysis, whereas tires were more commonly used for retinal holes; however, this reflects surgeon preference. Patients in the sponge group had a higher proportion of macula‐off eyes, resulting in worse preoperative BCVA. SSAS and ΔBCVA improvement were similar between both groups. Postoperative BCVA was worse in the sponge group both overall and in macula‐off eyes, likely due to poorer preoperative BCVA. With regard to explant complications, sponges showed higher rates of extrusion and required removal more often than tires.

## AUTHOR CONTRIBUTIONS

K.J. and P.K. conceived and designed the study; M.L. acquired the data; P.K. performed the statistical analysis and drafted the manuscript; all authors critically revised the manuscript and approved the final version.

## FUNDING INFORMATION

No specific funding was received for this work.

## CONFLICT OF INTEREST STATEMENT

The authors declare no competing interests.

## ETHICS STATEMENT

The study adhered to the tenets of the Declaration of Helsinki. As a retrospective analysis of data collected during routine clinical care, formal Research Ethics Committee approval was not required.

## Data Availability

The data underlying this article are available from the corresponding author upon reasonable request.
